# A Comprehensive Review: Current Strategies for Controlling Borer Pests During Grain Storage

**DOI:** 10.3390/foods15030473

**Published:** 2026-01-29

**Authors:** Muxi Zhong, Yujie Lu, Zhaojing Li, Md Mehedi Hassan, Shams Fawki, Tianyu Sha, Nyirahirwa Rebecca, Fei Liu, Peihuan He, Tao Zhang

**Affiliations:** 1 School of Grain Science and Technology, Jiangsu University of Science and Technology, Zhenjiang 212003, China; 2 National Research Institute of Food and Fermentation Industries Corporation Limited, Beijing 100015, China; 3 College of Ocean Food and Biological Engineering, Jimei University, Xiamen 361021, China; 4 Entomology Department, Faculty of Science, Ain Shams University, Cairo 11566, Egypt; shfawki@sci.asu.edu.eg; 5 Jiangsu Provincial Engineering Research Center of Grain Bioprocessing, Jiangsu University of Science and Technology, Zhenjiang 212003, China; 6 Academy of National Food and Strategic Reserves Administration, Beijing 100037, China

**Keywords:** borer pest control, influencing factors, physical methods, chemical methods, biological methods, emerging green methods, eco-friendly technologies

## Abstract

Grain storage is a critical part of preventing food crises and ensuring global food security. The stored grain is susceptible to infestation by borer pests, which not only degrade grain quality but also pose safety risks such as fungal contamination. Consequently, the control of borer pests represents a highly practical research direction in the food field. This review summarizes advances in stored-grain pest control technologies, covering physical, chemical, biological, and green methods. It highlights the advantages of cold plasma for degrading chemical residues via oxidative stress and nanocarriers for stabilizing pesticides, while exploring the development prospects of smart devices in precision control. Significant references are provided for developing new, highly efficient pest control technologies and systems to guarantee grain quality and safety.

## 1. Introduction

Grain storage is a crucial component in ensuring food security, and these stored grains play a vital role in guaranteeing food supplies during emergencies. Meanwhile, by conducting timely grain purchase, storage, and market distribution, they efficiently regulate the balance between supply and demand at the policy level, and thus become an important “cornerstone” for maintaining social stability. Therefore, many countries around the world are continuously increasing their grain storage.

However, grain storage worldwide faces the risk of loss, particularly due to storage pests, posing a serious threat to food safety. Approximately 8 to 14% of grain in the world is lost due to pests [1]. Typical borer pests such as *Sitophilus oryzae*, *Sitophilus zeamais*, *Callosobruchus maculatus*, and *Callosobruchus chinensis* have larvae and adult stages that feed on the interior of a grain. This feeding behavior causes the kernels to become hollow and produces excrement with an unpleasant odor. It not only significantly reduces the quality and nutritional value of the grains, but also leads to severe economic losses. Under favorable conditions in certain regions, the weight loss of stored grain caused by *S. zeamais* can surge from 11.25% to 35.12% within a storage period of three to six months [2].

To ensure food and grain safety, various technologies (physical, chemical, and biological methods) are employed to control stored-product borers. Physical methods mainly encompass preventing and controlling the presence of pests through temperature regulation, atmosphere adjustment, ionizing radiation, and inert powdering. Chemical methods include fumigants, protectants, and green plant essential oils. Biological approaches utilize natural enemies, pathogens, pheromones, growth regulators, and gene interference. However, the control of borers faces challenges such as pest resistance and pesticide residues. Consequently, researchers are actively exploring green, ecological, and sustainable strategies to mitigate these food safety risks.

Adequate equipment is the core support for pest management during grain storage, and its application is mainly concentrated in the field of physical and chemical control. In terms of physical strategy, food-grade inert powder is widely used, but ensuring uniform dispersion of the agent is still the focus of research and development to improve the effect of this technology. The new inert powder sprayer uses the electrostatic adhesion principle to achieve uniform diffusion of inert powder agents. Fumigation is the most commonly used chemical strategy in storage scenarios. Fully airtight warehouses are key to this process. By relying on the structural integrity of the bin for physical sealing instead of conventional plastic sheeting, these facilities enable effective full-volume fumigation. The combination of physical and chemical tools significantly boosts the reliability of pest management during grain storage.

## 2. Physical Control

### 2.1. Temperature Controlling Strategy

#### 2.1.1. High-Temperature Prevention and Control Technology

To reduce food safety risks, thermal control employs distinct strategies in various scenarios with heat transfer methods and energy sources. Solar drying is the simplest method, based on the thermal effect of solar radiation to raise the temperature of grain piles and act on borers. Commonly, grains are subjected to temperatures ranging from 45~65 °C and held within this thermal window for roughly two hours. Complete inhibition of adult eclosion in *C. maculatus* is achieved when the pest is subjected to temperatures between 57.4~64.1 °C for this specific duration [3]. However, it is a heavily weather-dependent method and struggles to achieve a uniform temperature distribution. Thus, it is only suitable for small-scale emergency treatments and use in small private production of grain. For household storage, boiling water immersion rapidly denatures proteins in borer pests like *C. maculatus* within minutes. This method is simple to operate and does not require specialized equipment.

The hot-air drying method achieves dual objectives of grain moisture reduction and pest control. The hot-air pest control technique involves continuously injecting 50~60 °C hot air generated by steam, gas, or electric heaters into grain storage silos or inter-pile voids for 30 s~36 h. This method penetrates deep into grain piles to thoroughly eliminate eggs and larvae. It leaves no chemical residues, but heat distribution depends on the grain pile density and micro-airflow and could be unequal. Notably, localized overheating or insufficient heating poses a risk of damage to heat-sensitive electrical equipment within the storage facility [4].

The molecular mechanisms underlying the effects of high temperature in pests remain incompletely explained, with the existing research primarily focusing on three pathways: the cellular microenvironment, protein homeostasis, and enzyme activity. Elevated temperatures cause cellular dehydration and an increase in the concentration of small molecules (such as K^+^ and Ca^2+^) in the hemolymph. This leads to alterations in the charge states of macromolecules, thereby affecting their function and molecular structure. In high-temperature environments, insects express heat shock proteins (HSPs) to enhance thermotolerance. Furthermore, heat stress induces insect autophagy to accelerate cellular structural renewal and clear protein aggregates and long-lived proteins, thereby improving heat resistance [5]. Temperature exceeding the pest’s optimal range also impacts its enzyme activity. High temperatures cause the inactivation of acetylcholinesterase, impairing neural transmission. Trehalose is a ubiquitous carbohydrate in insect hemolymph. Under elevated temperatures, expression of the trehalose synthase gene rdtps1 significantly decreases, leading to markedly reduced trehalose levels and decreased pest survival rates [6].

#### 2.1.2. Low-Temperature Control Technology

Low-temperature control technology is also an effective way to ensure food safety. Based on the cooling method and implementation environment, low-temperature control technologies can be broadly categorized into natural low-temperature utilization and mechanical refrigeration. The former is further subdivided into open-air freezing and natural ventilation methods. These technical approaches exhibit distinct differences in terms of applicable scenarios, control precision, and cost investment, thereby providing flexible, adaptable solutions for low-temperature pest control across diverse ecological regions and storage conditions.

While natural low-temperature technology is energy-free and economical due to its use of ambient cold, its practical application is heavily reliant on specific regional climates. Open-air freezing relies on environmental coldness during cold seasons. Grains are spread in thin layers on ground surfaces maintained at −10 °C to −5 °C. After 1~3 days/nights of deep freezing, the grains are promptly stored while cold. This method is simple and energy-free but constrained by weather conditions and site scale [7]. Natural ventilation method utilizes natural convection created by diurnal temperature variations, introducing external cold air through open doors and windows to gradually lower the storage temperature. Summer silo trials revealed that when warehouse temperatures dropped below 17 °C, the reproduction rate of *Rhyzopertha dominica* significantly decreased while grain moisture content remained virtually unchanged. This indicates that the technology inhibits the population growth of borer pests while preserving grain quality, making it suitable for storage scenarios characterized by significant temperature differences between the interior and exterior of the warehouse and controllable ambient humidity [8].

When ambient temperatures fail to provide sufficient cooling, mechanical refrigeration becomes a necessary supplement, i.e., utilizing cold air generated by refrigeration equipment to lower grain temperatures, thereby inhibiting borer development. Apart from temperature, a very important environmental factor for pests in stored grain is humidity, i.e., the combination of these two factors. *Sitophilus oryzae* survival rates vary under different temperature and humidity conditions. At constant humidity, mortality increases exponentially as temperature decreases. Within the temperature range of −5 °C to −25 °C, the mortality rate of pests at 40% relative humidity (RH) was consistently higher than that at 60% RH. Both the 40% RH-associated pest mortality rate and the 60% RH-associated pest mortality rate showed an upward trend as temperature decreased, until they leveled off at −25 °C [9]. This implies that mechanical refrigeration surpasses natural limitations and offers better, more precise temperature control than natural methods, rendering it applicable for storage situations with inadequate ambient cold or high precision requirements.

The mechanism of low-temperature pest control exhibits multi-pathway and multi-target characteristics, and a unified explanatory framework has yet to be established. Initial cold exposure alters cellular salt concentration and osmotic pressure, inhibits metabolism and active ion transport, leading to membrane depolarization and ion imbalance. Ultimately, this triggers apoptosis signaling through the opening of Ca^2+^ channels and activation of related enzymes [10]. Some other researchers think that as the temperature approaches the membrane lipid phase transition point, intracellular saturated fatty acid mobility diminishes, slowing transport and impeding growth processes [11]. Should temperatures further drop below freezing, intracellular ice crystals form. Their mechanical puncturing effect directly compromises membrane integrity, triggering irreversible death. Note that the initiation and intensity of these mechanisms are modulated by factors like stage sensitivity, humidity, acclimation [9], and storage scale. Differences in these aspects can alter the susceptibility of borers to low-temperature stress, ultimately impacting control outcomes.

### 2.2. Atmosphere Controlling Strategy

The controlled atmosphere strategy achieves the postponement of grain aging and the inhibition of borer pests and mold hazards by artificially modifying the gas composition among the grain kernels in the silo. The methods, such as N_2_, CO_2_, and O_3_, vary in equipment needs [12]. Their efficacy depends on temperature, humidity, gas composition, and pest stage. Higher temperatures and lower humidity accelerate mortality, while lower oxygen levels enhance lethality. Notably, Coleoptera pupae and eggs are the most tolerant stages, whereas adults are the most susceptible [13].

#### 2.2.1. Nitrogen Method

When N_2_ is added to the storage environment, borers suffocate and perish due to oxygen deficiency. High-purity nitrogen has a relatively strong controlling effect on phosphine-resistant borer populations. When the treatment duration reaches 10~20 days, a nitrogen (N_2_) concentration between 99% and 100% can achieve complete control of *Sitophilus granarius* and *C. chinensis* at all life stages, with the 99% concentration yielding the best effect. *Sitophilus oryzae* was exposed to a 99% nitrogen (N_2_) atmosphere for 2.5, 3, and 9 days, respectively. The results showed that the complete mortality of parental *S. oryzae* was only achieved when exposed at 28 °C for 3 days. Additionally, the fertility rate of the offspring was lower under long-term exposure conditions [14]. For the nitrogen charging mode using airbags, when the nitrogen concentration reaches 98%, it effectively hinders pest infestation [15].

#### 2.2.2. Carbon Dioxide (CO_2_) Method

Unlike nitrogen, CO_2_ is effective at moderate concentrations and requires shorter durations, making it more suitable for large-scale, efficient storage. When carbon dioxide concentrations are set to 40%, 60%, and 80%, adult *R. dominica* require 8 days, 6 days, and 4 days of exposure, respectively, to achieve a 100% mortality rate [16]. At 25 and 50% CO_2_, pests predominantly alter insulin metabolism in response, while at 75 and 95% CO_2_, pests predominantly alter the metabolism of trehalose (increased expression of TRE1-2, TRE1-3, and TPS) [17].

In addition, the combination of low oxygen and high carbon dioxide concentrations has an N_2_ additive effect. When comparing the survival of *C. maculatus* under two modified atmospheres, the mortality rate of *C. maculatus* is higher in the environment containing 18% CO_2_ than in the one without CO_2_, and the surviving borers grow faster [18]. High CO_2_ concentration lowers the pH of insects’ hemolymph, leading to impaired cell membrane function; enzyme denaturation; reduced reductions in reduced coenzyme II (NADPH) and glutathione, reduced cholesterol, lipid, and fatty acid synthesis; and potentially toxic release of reactive oxygen species [19,20,21].

#### 2.2.3. Controlled Atmosphere with Hypoxic

Hypoxic storage is a popular method for food preservation. The mortality rate of *S. oryzae* reached 90% within 4 days at an O_2_ concentration of 1%, and after 10 days at an O_2_ concentration of 3%. The lower the O_2_ concentration, the shorter the time required for *S. oryzae* to die. Additionally, *S. oryzae* cannot develop into adults under O_2_ concentrations of 1% and 3% [22].

Insects’ metabolism self-regulates differently under different low oxygen concentrations. Specifically, exposure to oxygen-deprived conditions disrupts physiological homeostasis, suppressing oviposition and adult emergence [23]. At moderate levels of hypoxia, insects regulate the process by increasing respiratory metabolic activity, proliferating cells in the tracheae, and inducing the transcriptional regulation of hypoxia-inducible factors [24,25,26]. At higher levels of hypoxia, insects will reduce the consumption of energy [27]. When anaerobic metabolism is carried out in *R. dominica*, the concentration of lactate increases, and the activities of cytochrome c oxidase and mitochondrial uncoupling are reduced to one-third of their original levels [28].

#### 2.2.4. Controlled Atmosphere with Ozone (O_3_)

The ozone fumigation method has a strong borer control efficacy. Ozone can be produced by passing an electric arc through air. This method requires no chemical storage or container handling and results in natural decomposition with zero residues. It can completely kill *Echinocnemus squameus* in grain storage facilities after only 2 days of exposure, even at concentrations ranging from 0 to 70 ppmv (parts per million by volume) [29]. Treating *C. maculatus* in cowpeas inside a fumigation chamber shows that the weevils at the bottom are more affected than those at the top. Both 45.52 mg/g and 65.97 mg/g are required to kill 50% of adult weevils at the bottom and top, respectively, with no impact on cowpea germination [30].

### 2.3. Inert Powder Strategy

Inert powder is a food additive of an anticaking agent with silica as the main component. At present, the explanations for the principle of its pest control effects are as follows: When inert powder approaches the pores on the pest’s surface, it absorbs the pest’s body fluids through van der Waals forces. When the inert powder accumulates on the pest’s surface, it pierces the pest’s intersegmental membranes, accelerating the loss of body fluids. In addition, it stimulates the pest’s sensory system and increases its movement frequency.

The effectiveness of inert powder against the stored grain borer is related to its dosage, performance, and type. When silica powder with higher silica content, lower pH value, and stronger oil absorption is used to control *S. oryzae*, it achieves better efficacy—a smaller amount of such powder applied within a certain period can result in complete mortality of the pest [31]. Diatomaceous earth, gypsum powder, and basalt powder all belong to inert powders. When comparing the dosage and number of days required for the three inert powders to completely kill *S. zeamais*, diatomaceous earth had the best lethality—a dosage of 0.05 g/20 g of corn achieving 100% mortality in 11 days. Comparatively, gypsum powder required a dosage of 0.2 g/20 g of corn and acted for 21 days, while basalt powder needed a dosage of 0.2 g/20 g of corn and acted for 28 days [32].

### 2.4. Electromagnetic Wave Strategy

An electromagnetic wave is a promising method for gaining safety. Insects have a dielectric heating effect. Their bipolar molecules tend to align with the magnetic field and rub against the resistance generated by their rapid movement, causing heat dissipation in insects exposed to the microwave [33]. Once the microwave energy density exceeds 0.0325 kWh/kg, 100% of borers are dead. However, microwave efficacy is influenced by moisture content, which limits its ability to achieve uniform treatment in bulk grain [34]. An electromagnetic wave with a frequency exceeding 100 kHz is known as radio frequency, which heats materials by converting absorbed electromagnetic energy into thermal energy [35]. When using a 6 kW, 27.12 MHz radio frequency, heating is maintained at 52~54 °C, the borer mortality rate increases with the increase in temperature, and 100% mortality is achieved by heating at 54 °C within 12 min, demonstrating its superiority in the uniform treatment of bulk grain [36].

## 3. Chemical Control

### 3.1. Fumigation Strategy

#### 3.1.1. Fumigants

Fumigants (Table 1) are widely employed. Among them, the more common ones are phosphine and sulfuryl fluoride, as presented in Table 1. Phosphine is significant in the control field due to its broad insecticidal spectrum, rapid effect, low cost, and no residue [37]. Phosphine fumigation of grain silos can achieve 100% borer mortality on the surface of grain piles under conventional conditions [38]. It cannot be overlooked that the excessive use of phosphine has led to an increase in pest resistance [39]. This has driven the demand for research into alternative fumigants and optimized application strategies. Fumigants differ in diffusivity, scope, and safety. Thus, practical evaluation must integrate these attributes rather than relying solely on mortality data.

#### 3.1.2. The Principle of Fumigation

**Figure 1:**
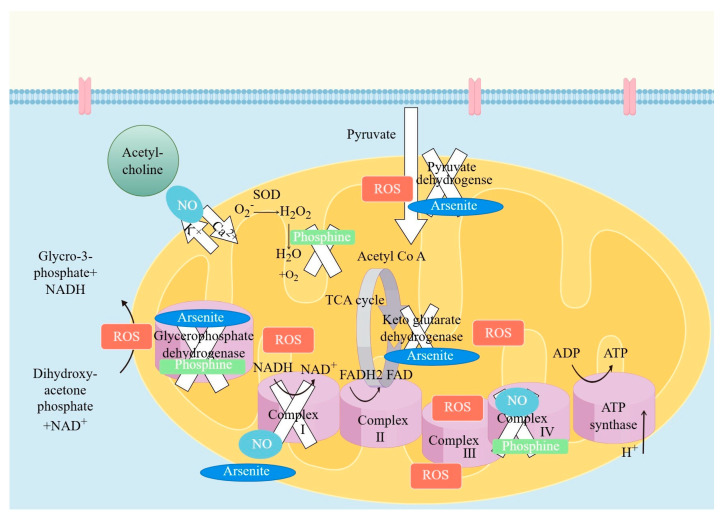
The principle of phosphine in insecticidal use [43].

The principle of fumigation will be explained using the example of phosphine. Phosphine enters the pest’s body via its valve or epidermis. Currently, there are three explanations regarding the principle of pest control (Figure 1): Exposure of pests to phosphine increases the intracellular content of reactive oxygen species, which is involved in oxidative phosphorylation; when phosphine is used as a medium, it reduces cytochrome oxidase activity, disrupts electron transport in mitochondria, and leads to cell death. Phosphine affects the activity of dihydrolipoic acid amide dehydrogenase, which then influences the production of coenzyme I (NADH), resulting in the inhibition of aerobic respiration of pests. Additionally, phosphine inhibits the activity of acetylcholinesterase and increases the content of acetylcholine, leading to nervous system dysfunction [43].

A study suggests the principle behind using sulfuryl fluoride to control pests: The fluoride ion in sulfuryl fluoride is toxic, while the magnesium ion has enzyme activity. When these two ions combine, they destroy the enzyme’s spatial structure, disrupting glycolysis and inhibiting the metabolic cycle of fatty acids and glycolysis. This ultimately leads to the depletion of the essential energy required for insect survival, causing their death [44]. Due to its distinct mode of action compared to phosphine, sulfuryl fluoride serves as a viable alternative for resistance management.

#### 3.1.3. Fumigation Techniques and Applications

Effective fumigation relies on sustaining lethal concentrations and ensuring uniform distribution. However, these two core factors are further influenced by the distance of borer pests from the fumigation source, their developmental stage, and the application method, which in turn dictates the overall success of the treatment. The mortality rate of *S. granarius* is inversely related to its distance from the fumigation point: the mortality rate reached 100% at 0 m, while at 5 m, 10 m, and 15 m, the mortality rates are 11.67%, 3.33%, and 1.67%, respectively [45]. To mitigate the risk of sub-efficacy in remote zones of large-scale bulk storage, optimizing gas distribution uniformity is essential for effective fumigation process control.

In terms of application strategy, cyclic fumigation can reduce the risk of pest survival caused by localized low-concentration zones by improving gas transport within the grain bulk. Cyclic fumigation with a thermosiphon system maintained phosphine concentrations above the effective insecticidal concentration (200 ppm) from the 4th to the 106th day of fumigation [46]. This demonstrates that for bulk grain or large-volume grain masses, the duration of effective concentrations and uniform distribution are more critical than short-term peak concentrations.

Phosphine has different toxicities to bores at various developmental stages. The higher the developmental level, the greater the sensitivity to phosphine, with adult borers being the most sensitive [47]. Therefore, application strategies must target the tolerance of the most resistant stages rather than just adult mortality. Engineering solutions like cyclic fumigation, extended exposure, or multi-point dosing are essential to address borer pest crypticity and mitigate resistance risks.

### 3.2. Protection Agent Strategy

#### 3.2.1. Grain Storage Protective Agents

Grain storage protective agents are a category of insecticides mixed into the grain kernel to prevent pests from causing damage through the contact-killing effect. Their insecticidal range and resistance risk are jointly determined by their physicochemical properties and sites of action. Currently, approved grain storage protective agents in China include Fenitrothion, K-Obiol, etc. [40]. (Table 2).

#### 3.2.2. The Insecticidal Principle of Protective Agents

**Figure 2:**
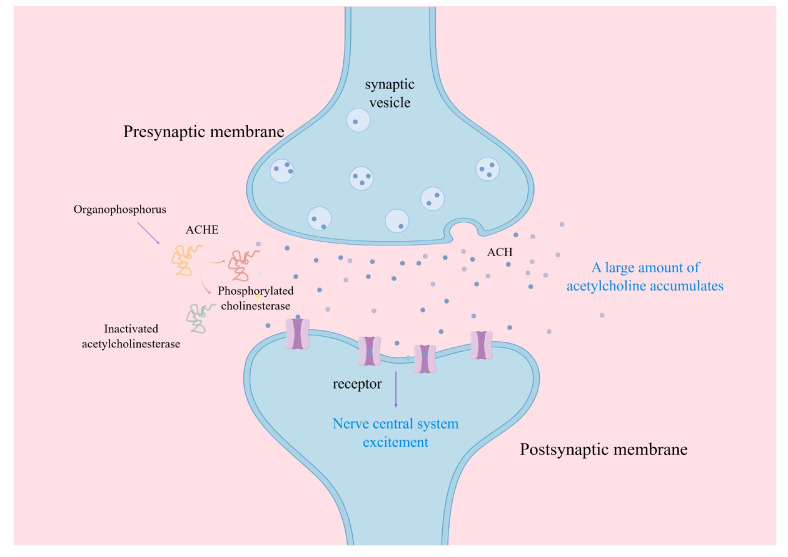
The principle of organophosphorus insecticides [48].

Organophosphorus is a commonly utilized protective agent for grain storage. It is lipid-soluble and diffuses quickly after absorption by acetylcholinesterase transporter-containing membranes, releasing acetylcholine and blocking nerve signal transmission. A significant amount of accumulated acetylcholine stimulates the central nervous system and its effector organs—the physiological functions of the tissues and organs are altered, generating toxicological symptoms (Figure 2). While their combined lipophilic and hydrophilic properties facilitate deep penetration into the grain bulk, these properties can also cause maldistribution or inadequate exposure times due to rapid diffusion, rendering them prone to resistance development under prolonged application [48].

#### 3.2.3. Insecticides’ Techniques and Applications

Different insecticides vary in borer control efficacy depending on the system of application. Pirimiphos-methyl, malathion, deltamethrin, and the deltamethrin-(S)-methoprene mixture are common protectants. When adult *S. oryzae* were exposed to these four protectants at different concentrations, the LC_50_ values revealed toxicity differences—pirimiphos-methyl was the most toxic, followed by malathion and deltamethrin, with the deltamethrin-(S)-methoprene mixture being the least toxic [49]. However, these LC_50_ values must be comprehensively evaluated in conjunction with the current status of insect resistance and the application system.

Since *S. oryzae* typically exhibits stronger resistance to malathion than to pirimiphos-methyl [49], and due to the rapid diffusion of organophosphates, application strategies must prioritize the uniform distribution and persistence of the agent within the grain bulk. Deltamethrin has diverse effects when applied to different surfaces of the grain storage environment. When 100 mg/m^2^ deltamethrin emulsion is applied to non-porous plastic and porous concrete surfaces, 100 percent borer mortality is achieved after 1 day on non-porous surfaces, while on porous concrete surfaces, adsorption leads to a reduction in the effective contact dose, thereby limiting its effectiveness [50].

Volatile fatty acids are environmentally friendly and have good insecticidal effects. Volatile fatty acids (VFAs) are the products of the degradation of organic matter (hemicellulose and cellulose) by anaerobic and facultative bacteria during acidification. The application of VFA to *C. maculatus* reveals a positive correlation between the mortality and the concentration of VFA. Among them, 4 μL of propionic acid and 8 μL of butyric acid have the strongest lethal effect on *C. maculatus*. The vast majority of VFA has a significant anthelmintic effect [51]. Nevertheless, the volatility of VFAs constrains their residual activity, positioning them primarily as a supplementary tool. Consequently, the scientific application of grain protectants necessitates the integration of environmental factors—including diffusion, exposure duration, and adsorption. By employing strategic formulation combinations, this approach mitigates resistance risks and improves overall control performance.

#### 3.2.4. Factors Influencing the Efficacy of Protective Agents

In grain storage systems, besides the type of agent, dosage, and application surface, the factors influencing the efficacy of protective agents encompass the application method, storage conditions and their sensitivity, pest density, type of grain, and grain moisture content [52].

### 3.3. Plant Essential Oil Strategy

Common spices, such as lemon, mint, star anise, chili peppers, garlic, and ginger, have a certain inhibitory effect on grain borers and are pure natural green products with advantages that other chemical agents cannot compare with (Table 3). At its core, the insecticidal potency of plant essential oils is highly dependent on the integrated interaction of component characteristics, action mechanisms, and environmental variables.

#### 3.3.1. The Insecticidal Principle of Plant Essential Oils

As an example, we will explain the functioning of the basil essential oil, which can be used to control pests. Linalool and 4-allylbenzylphenol in the essential oil of basil possess strong fumigant and contact toxicity. On the one hand, they penetrate the pest’s body through spiracles and the body wall, demonstrating excellent gas diffusion capabilities. On the other hand, 4-allylbenzylphenol and 1,8-cineole inhibit the activity of the acetylcholinesterase enzyme, which is implicated in catalyzing the hydrolysis of neurotransmitters at neuromuscular and synaptic junctions, thereby inducing the interruption of nerve conduction and physiological disorders. Thus, 4-allylbenzylphenol also curbs the oviposition rate of adult pests [58,59]. Secondly, β-esterase and alkaline phosphatase regulate the alkaline balance within organisms and participate in diverse physiological processes. Basil can reduce the activities of both enzymes [60]. This multi-modal mechanism of action reduces the risk of pests developing single-target resistance.

The presence of 1,8-cineole, a monoterpene in the essential oils of laurel, rosemary, lavender, and thyme, enters the respiratory system by fumigation, the body through direct contact via the stratum corneum, and the digestive system by ingestion [59].

#### 3.3.2. Essential Oils—Insecticidal Techniques and Applications

The insecticidal effect of plant essential oils is associated with the plant source composition, the dose, the borer species, temperatures, and light conditions. Monoterpenes are present in plant essential oils that have considerable borer control and repellent effects. Monoterpenes exhibit different fumigation effects depending on the borer species, with better control effects on *C. maculatus* than *S. zeamais*. In the case of geraniol, citronellol, and citronellal, a dose of up to 8 µL is necessary to achieve a high lethality rate in *C. maculatus*, whereas for eugenol, up to 4 µL achieves the same effect [61].

The efficacy of plant essential oils varies under different temperatures and light conditions, and basil essential oil is effective against *S. zeamais* below 20 °C and in the absence of light [62]. This environmental sensitivity further highlights the need to balance the diffusion advantages and persistence limitations imposed by volatility. Therefore, their application must integrate physicochemical stability and environmental adaptability, which can be optimized through formulation strategies to balance resistance management and temporal control.

## 4. Biological Control

### 4.1. Predatory and Parasitic Natural Enemies Strategy

Predators prey on specific species and developmental stages of borers, and sometimes are cannibals. They primarily pierce the prey and inject digestive fluids or saliva to decompose the internal tissues of insects, and then consume their bodily fluids. Parasites attach themselves to other animals internally or externally for some time or throughout their lifespan, and subsist by extracting nutrients from the host. The majority of parasites can paralyze their hosts, thereby inhibiting their growth. The paralyzed hosts are incapable of continuing to feed and cause damage to stored products [40].

Both the type of natural enemies and the morphology of pests are influential factors in the effectiveness of predatory and parasitic pest control. *Xylocoris flavipes* is a predator, and *Theocolax elegans* is a parasitic wasp. When *X. flavipes* is utilized independently, the survival rate of the offspring of *S. oryzae* and *R. dominica* is 65% and 1.6%, respectively. When *T. elegans* is independently employed, the survival rate of the offspring of *S. oryzae* and *R. dominica* is 41% and 1.6%, respectively, and when combined control is implemented, the survival rate of offsprings of *S. oryzae* and *R. dominica* is only 0.3 and 0.4%, respectively. The synergistic effect of combined control is highly evident [63]. The combined control by low temperature and parasitic natural enemies is also satisfactory—when *S. granarius* was co-cultured with *Lariophagus distinguendus* at 16 °C, 18 °C, and 20 °C, its mortality increased with temperature, reaching 70% at 20 °C [64].

### 4.2. Pathogen Strategy

Insect pathogens employed in biological control are infectious to borer pests. Entomopathogenic methods can reduce the utilization of chemicals. These pathogens are solely transmitted among pests, and are disseminated in four principal manners: feeding on food containing pathogens, contact with deceased and diseased insect carcasses and their feces during feeding or exercise, contact during mating, and transmission by eggs from the female adults to their offspring. Insect pathogens can control *S. granarius* and *S. oryzae*. After 7 days of treatment by entomopathogenic fungi at 20 °C and 25 °C, *Isaria fumosorosea* and *Metarhizium anisopliae* (1 × 10^8^ spores/mL) showed the most significant effect on *S. granarius* (mortality > 80%), while *M. anisopliae* and *Beauveria bassiana* were most effective on *S. oryzae* (mortality > 85%) [65].

### 4.3. Pheromones and Growth Regulators Strategy

Pheromones are natural compounds secreted by specific insects, serving as a bridge for intraspecific communication and influencing insect behaviors like aggregation, habitat marking, and mating. They include aggregation pheromones, sex pheromones, oviposition inhibitors, and volatiles released by dampened grains [66]. Pheromones can serve as cores in insect traps. When pheromones are used in combination with biological agents, borers are lured into traps by the pheromones, and upon contact with the biological agents, they carry these agents back to their colonies, spreading the epidemic [67].

Insect growth regulators control and regulate the growth and development of insects. They typically act on larvae by influencing their development to reduce the reproduction of offspring. For instance, juvenile hormone analogs (JHA) retard embryonic development by affecting pupal formation, reproduction, and midgut cells. When 4 mg/kg pyriproxyfen is utilized, the incidence of borer pests is less than 16% at week 0 and no more than 40% by week 12 [68].

### 4.4. RNA Interference (RNAi) Strategy

**Figure 3:**
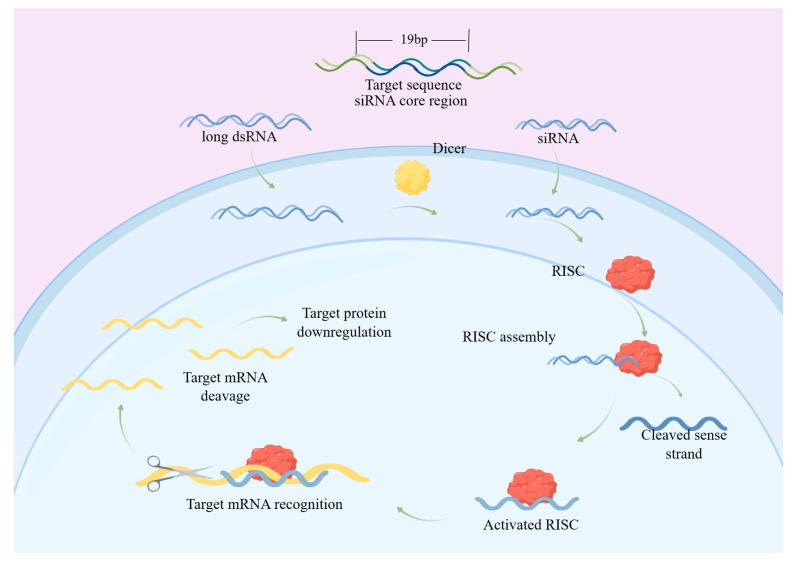
The mechanism of RNAi action [69].

Constrained by limited commercial adoption, RNAi remains an auxiliary synergistic tool rather than an independent strategy, a constraint rooted in its unique mechanism of action—the technology does not take effect through direct physical contact, but rather it is used to silence specific gene expression via small interfering RNA molecules for normal development or physiological activities of pests. The specific principle of prevention is as follows: endogenous mi-RNA becomes functional miRNA after transcription and secondary processing, and then silences specific genes through two pathways. One is that miRNA does not fully adhere to the Watson–Crick base pairing rule and inhibits the translation of the targeted mRNA in the non-coding region at the 3′ end. The other is that the endogenous or exogenous si-RNA guideline chain is reorganized in the RISC (silencing complex), causing sequence-specific degradation of the target mRNA (Figure 3) [69].

Application methods include feeding, injection, spraying, and soaking. RNA interference (RNAi) can be induced by feeding dsRNA to borer pests. Terpinene-4-ol is highly insecticidal to *S. zeamais*, and feeding the weevil dsRNA knocks down NADPH-cytochrome P450 reductase, increasing its sensitivity to terpinene-4-ol [70,71,72]. To improve borer control efficacy further, nanoparticles (orally administrable to borers) improve RNAi efficiency by reducing dsRNA degradation and boosting intact dsRNA uptake; liposomes improve it as delivery carriers; and siRNA targeting can also be strengthened via chemical modification [73].

## 5. New Green Prevention and Control

While humans have long worked to secure food supply and created various technologies against stored-grain borers, there are still disadvantages. Additionally, new developing tendencies, such as green and sustainable requirements, are gaining attention. Researchers are also continuously exploring new pest control technologies (such as biopesticide Spinosad, cold plasma, and nanomaterials) to ensure food security.

### 5.1. Spinosad

Biopesticide Spinosad exhibits broad-spectrum insecticidal efficacy, low toxicity, low residue, and a unique insecticidal mechanism, thereby being recognized as a green and safe protective agent for grain storage. It is a metabolite of *Saccharopolyspora spinosa* that can influence acetylcholine and γ-aminobutyric acid receptors and thereby interfere with the physiological activities of insects. Borer species, dosage, surface of action, and temperature are factors that influence the control efficiency of control by Spinosad. Spinetoram (0.5 or 1 ppm) is more effective against *R. dominica* and *C. maculatus* than other borer pests [74]. Regarding the environment, when a low concentration of Spinosad is applied on the surface of ceramic floor tiles, its insecticidal activity is stronger than that on composite flooring and concrete [75]. The efficacy of Spinosad against S. zeamais and *S. granarius* showed a positive correlation with temperature. When the temperature reached 30 °C, 100% mortality of *S. zeamais* and *S. granarius* could be achieved [76].

### 5.2. Cold Plasma

**Figure 4:**
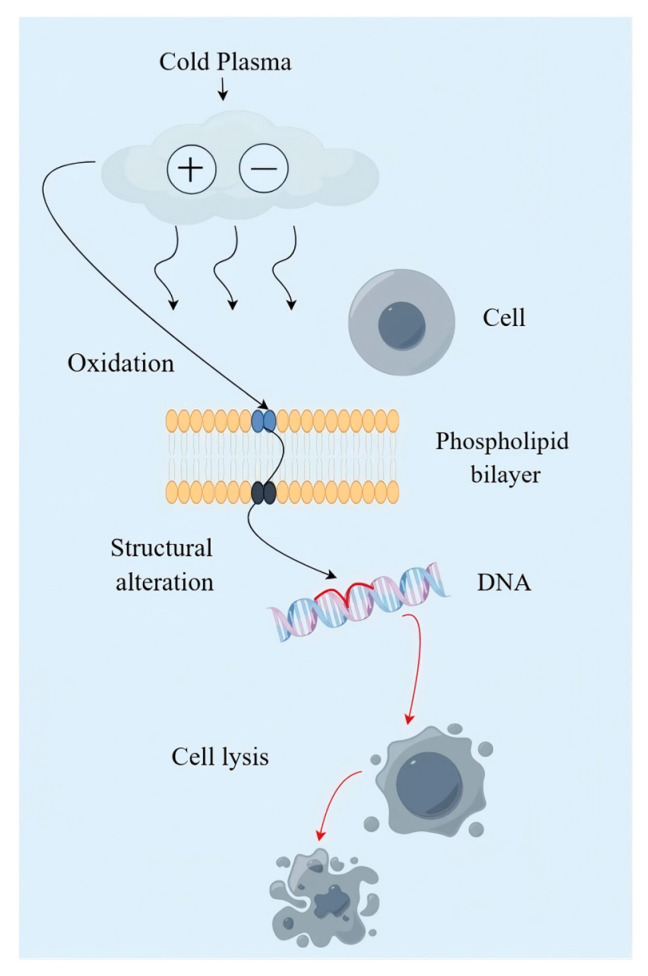
The mechanism of cold plasma action [78].

Cold plasma technology involves the interactions among free radicals, charged particles, nitrogen substances, reactive oxygen species, and vacuum ultraviolet light. Through signal transduction processes, cell destruction, and oxidative stress (an imbalance between oxidation and antioxidation), it reduces the activities of antioxidant enzymes [such as superoxide dismutase (SOD) and catalase (CAT)] that disrupt the metabolism of insects, resulting in their death (Figure 4) [77]. Plasma with 5% N_2_, 30% CO_2_, and 60% O2 can poison *C. maculatus* at atmospheric pressure: treated at 70 kV for 3 min, its larval and egg mortality exceeded 90% [78]. Noble gases are also insecticidal under high voltage. After exposure to Ar and He cold plasma with an input voltage of 24 kV, 2.5 kHZ, and 7.5 kHz for 30 to 120 s, the Ar cold plasma has a stronger insecticidal effect on *S. oryzae*. When exposed at 7.5 kHz for 30 s, *S. oryzae* can be completely killed after 1 h [77].

### 5.3. Nanomaterials

Nanomaterials are fabricated using materials like carbon, silicates, ceramics, metals, and semiconductor quantum dots. Nanoparticles, with sizes ranging from 1 to 100 nm, constitute different types of basic units of nanomaterials [79]. Nanoparticles induce an oxidative stress response in borers, disrupt the homeostatic balance within cells, damage the structural integrity of the borer organism, and simultaneously express a toxic effect on specific structures of the borer [80]. The direct application of nanoparticles on grain surfaces can cause the stored grain borer to die due to dehydration or decreased viability, like inert powders. For instance, the application of 2 g/kg of silica nanopowder on the surface of maize resulted in 100% lethality of four-grain storage borers, including *S. oryzae*, within one day.

Nanomaterials can also serve as carriers of pesticide compounds, which significantly enhances the stability of pesticides [80]. Nanoparticle pesticides exhibit high fumigant and contact toxicity against grain storage borers [81].

Chitosan is a biodegradable, antibacterial, and antioxidant natural polysaccharide derived from chitin [82]. Chitosan was employed as a nanoencapsulation material to encapsulate spinosyn and deltamethrin. Chitosan/pesticide nanoparticles were synthesized to achieve significant encapsulation within 6 h [83].

Nanomaterials, such as branched amphiphilic proteins, chitosan, liposomes, and quantum dots, can be utilized as nucleic acid carriers for controlling borer pests, which improves the delivery efficiency of novel nucleic acid insecticides. Treating *S. zeamais* in wheat with chitosan as a carrier for cytochrome c oxidase double-stranded RNA (dsRNA) can kill half of the adult insects after 10 days [84]. Although nanomaterials demonstrate significant efficacy in physical extermination and active ingredient delivery, their translation into granaries is constrained by high costs and dust hazards.

## 6. Comparison of Prevention and Control Methods

From the above-mentioned control methods for grain borers, popular approaches were selected from physical, chemical, and biological categories for comparison. As shown in Table 4, cold plasma, limited by complex technology, high cost, and narrow coverage, is better suited for small-scale or high-value grain storage. Phosphine fumigation applies to large-scale grain storage, with strict airtightness required. Plant essential oil fumigation fits household grain storage and early pest prevention. Entomopathogenic fungi work in specific temperature–humidity environments, as local control, or as an addition to other measures. Natural enemy control is ideal for long-term pest prevention or grain storage with strict chemical residue standards.

## 7. Prevention and Control Equipment

**Figure 5:**
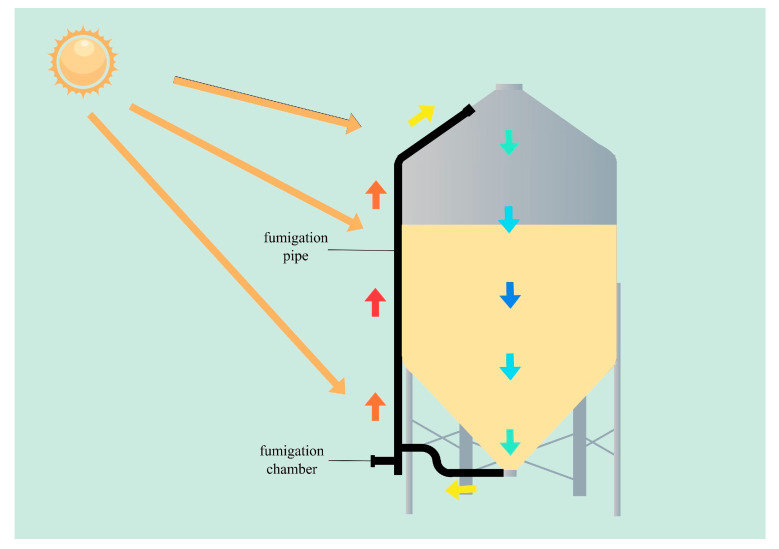
Thermosiphon device (black colored) [46].

For the chemical strategy, a thermosiphon device (Figure 5) for fumigation has been developed. It can be installed on silo exteriors. The thermosiphon device has a main body of a black 1-decimeter-diameter high-density polyethylene pipe connecting the silo’s upper and lower cones. A fumigation box at the lower end of the pipe’s vertical part allows ground-placed fumigants. Heated fumigant gas rises through the pipe into the silo top, cools among the grains to increase density, sinks to the silo bottom, and re-enters the pipe for cyclic fumigation (Figure 5) [46]. In addition, fumigable warehouses with excellent airtight design enable whole-store fumigation without tent equipment, controlling both warehouse and grain pests simultaneously while improving storage utilization [87].

For physical strategy, an inert dust spraying equipment with an electrostatic function was invented. It is constituted with an electrostatic generator, a powder injector, and a high-pressure air pump. Inert dust, pre-treated by the generator to gain electrostatic adsorption, is pressurized by the pump and sprayed onto grain on the conveyor belt, enabling tight bonding with grain via electrostatic adsorption before silo storage [88].

While current pest control equipment remains limited or traditional, it is evolving toward intelligent, precise, and green pest management. The precise and green pest management entails adopting the most suitable and eco-friendly intervention methods under precise monitoring, replacing traditional broad-spectrum and excessive application. Specifically, it involves point-to-point physical removal of specific pests in designated areas, targeted fumigation in pest-infested zones using intelligent gas delivery systems, or automatic, quantitative release of specific natural enemies or sex pheromones based on the monitoring results.

## 8. Summary

With food safety requirements increasing, borer pest control is entering a precise, green, and controllable new stage. This paper analyzes research progress of three mainstream control groups of methods: physical control uses modified atmosphere or ionizing radiation to reduce borer populations; chemical control implements with quantitative release of fumigants and plant essential oils; and biological control realizes targeted interference via natural enemies, pheromones, and pathogens. Additionally, we compared their mechanisms, effects, key restricting factors, and applicable scenarios. Focusing on three emerging green technologies (Spinosad, plasma, and nanomaterials), the study clarifies their action targets, formulation processes, and applications. Equipment for precise and eco-friendly strategies of control equipment can realize on-demand pesticide application and on-site pest elimination. Notably, the combination of green technologies and intelligent equipment would minimize pesticide dosage while preserving grain quality.

## Figures and Tables

**Table 1:** List of common fumigants and their characteristics, advantages, and drawbacks.

Fumigant	Characteristics	Advantages	Drawbacks	Reference
Phosphine	Colorless and odorless poisonous gas, reductive, and easily photolyzed.	Wide insecticidal spectrum, high efficacy, and low cost.	Widespread resistance, flammability, and prolonged exposure likely lead to bronchitis and dyskinesia.	[40]
Sulfuryl fluoride	Colorless and odorless gas at room temperature and pressure, reacts with water, and is sparingly soluble.	Wide insecticidal spectrum, strong penetration, high stability, low toxicity, and weak adsorption to grain.	High cost, weak ovicidal effect, and easy to form a strong greenhouse effect.	[40]
Methyl bromide	Colorless gas, and low boiling point.	Wide insecticidal spectrum, strong diffusion, strong penetration, wide temperature range, wide range of applications, highly irritating, and bactericidal.	Increases the Earth’s ultraviolet light due to the depletion of substances in the ozone layer, with high environmental and human impacts.	[41]
Ethylene dibromide	Transparent colorless liquid, slightly soluble in water, and non-flammable.	Widely used as waterproofing products, water purifying agents, and solvents.	Carcinogenic and toxic to reproduction, affecting the central system and even causing pulmonary edema and bronchitis by inhalation.	[42]
Ethyl formate	A colorless liquid that is slightly soluble in water and has an aromatic odor.	Biodegradable, non-corrosive, with a low boiling point and fast killing activity.	Flammable, excessive inhalation causes damage to the nervous and respiratory systems, and there is also a high cost.	[40]

**Table 2:** Characterization, advantages, and drawbacks of common protective agents.

Protective Agent	Characterization	Advantages	Drawbacks	Reference
Malathion	Colorless to light yellow oily liquid.	High efficiency, low toxicity, broad-spectrum, strong knockdown power, and short residual effect period.	Highly affected by temperature and pH, and no systemic action.	[40]
Fenitrothion	Brown-yellow oily liquid with mild garlic odor.	Rapid action, high efficiency, good persistence, a broad spectrum of insecticides, and stability in the presence of light.	Combustible when exposed to open flame and high heat, easily decomposed in high temperatures, and metal and alkaline media can lead to acute poisoning.	[40]
Pirimiphos-methyl	Brownish-yellow liquid.	Low toxicity, high efficiency, wide insecticide spectrum, easy biodegradation, long shelf life, and widely used.	Toxic to non-target organisms.	[40]
Deltamethrin	White or off-white crystalline powder, chemically unstable, and easily degradable.	Rapid action, strong knockdown force, repellent action, broad spectrum of insecticides, and strong penetration.	Exhibits poor stability in alkaline solutions and bleaching powder.	[40]

**Table 3:** The insecticidal activity of the components of plants.

Ingredient	Spices Containing This Ingredient	Characteristic	Insecticidal Activity	Principle of Insecticidal Action	Reference
Limonene	Chinese prickly ash	A colorless liquid that is insoluble in water.	Contact, fumigation, repellence, attraction.	(a) Damages the cuticle on the body surface, accelerates the loss of water, and triggers an oxidative stress response. (b) The electron transport chain in mitochondria is damaged, and the activity of related enzymes is decreased. (c) Sensory nerves and motor nerves spontaneously generate nerve impulses, leading to spasms, convulsions, etc. (d) The activity of acetylcholinesterase is decreased, resulting in neurotoxicity.	[53]
Linalool	Chinese prickly ash, ginger	A colorless liquid, flammable and fragrant.	Contact, fumigation.	(a) Acetylcholinesterase is inhibited, and acetylcholine accumulates in the synaptic cleft, producing neurotoxicity. (b) α-amylase is inactivated, and the development is hindered.	[54]
Anethole	Star anise	A colorless or slightly yellowish liquid or crystal.	Fumigation, repellence, attraction.	(a) A significant decrease in the activity of acetylcholinesterase triggers neurological dysfunction.	[55]
Capsaicin	Chili pepper	A white crystal, insoluble in water and liable to decompose at high temperatures.	Repellence, antifeedant effect, inhibits growth and development.	(a) Capsaicin stimulates the gustatory and olfactory organs of pests, reducing their food intake. (b) Capsaicin acts on the transient receptor potential vanilloid 1 (TRPV1) to cause a transformation in the physical properties of the biological membrane; it adheres to the lipid bilayer and changes the membrane fluidity to interfere with the thermoregulation of insects.	[56]
Citral	Ginger	A colorless or slightly yellowish liquid with a lemon scent.	Contact, fumigation, repellence; inhibits growth and development.	(a) Inhibit the signal transduction generated by the excitatory neurotransmitter octopamine, leading to problems such as energy consumption and hindered movement. (b) The activity of acetylcholinesterase is altered, resulting in neurotoxicity.	[57]

**Table 4:** Comparison of typical methods in physical, chemical, and biological prevention and control.

Method	Conditions and Effects	Advantages	Disadvantages	Reference
Cold Plasma	*S. oryzae* achieved 100% mortality under 24 kV voltage, 7.5 kHz Ar-He cold plasma, 30 s exposure time, and 1 h retention time.	No toxic residues, no impact on grain quality, and guaranteed worker safety	High equipment cost, limited coverage, and significant differences in pest stage resistance	[76]
Phosphine Fumigation	*S. oryzae* across regions reach 95% mortality at 24 h, 48 h, 72 h exposure times, with corresponding fumigation concentrations of 0.058~5.420 mg/L, 0.029~6.160 mg/L, 0.021~2.940 mg/L, respectively.	Wide application range (including pests in grains, legumes, tobacco, and other items), strong penetration ability (can penetrate packaging materials and some containers), comprehensive coverage, low cost, and simple operation	Pest resistance is increasingly strong, toxicity to non-target organisms, susceptibility to environmental stability, and long ventilation waiting time (generally 24~72 h)	[85]
Plant Essential Oil Fumigation	The mortality rate of *S. zeamais* exceeds 90% under the conditions of 2 mL/mL air of borage leaf essential oil and an exposure time of 144 h.	Low pest resistance, guaranteed worker safety, environmental friendliness, strong targeting, and high sustainability	Long onset time (only 42% mortality rate of *S. zeamais* at 48 h under the same concentration), short duration of effect, poor stability, and high cost	[86]
Entomopathogenic Fungi	*S. granarius* and *S. oryzae* reach 92.69% and 93.66% mortality, respectively, under 1 × 10^8^ spores/mL *Isaria fumosorosea* (for *S. granarius*)/*Beauveria bassiana* (for *S. oryzae*), 25 °C, 7d treatment, 75 ± 5% RH, and 16 h light/8 h dark cycle.	No chemical residues, strong targeting, high sustainability, and compatibility with multiple control methods (such as natural enemy and pheromone control)	Influenced by environmental temperature and humidity, slow onset of effect	[65]
Natural Enemy	*Bug Orius sauteri* is released two weeks before the wasp *Anisopteromalus calandrae*, and their combined release results in 99.7% mortality of *S. oryzae* offspring.	Covers the complete developmental stages of *S. oryzae*, high sustainability, reduces reliance on chemical agents, and strong adaptability	Slow onset of effect (control period is 6 weeks), and challenges in the controllability of natural enemy insects	[63]

## Data Availability

No new data were created or analyzed in this study. Data sharing is not applicable to this article.
